# JPlogP: an improved logP predictor trained using predicted data

**DOI:** 10.1186/s13321-018-0316-5

**Published:** 2018-12-14

**Authors:** Jeffrey Plante, Stephane Werner

**Affiliations:** Lhasa Limited, Granary Wharf House, 2 Canal Wharf, Leeds, LS11 5PS UK

## Abstract

The partition coefficient between octanol and water (logP) has been an important descriptor in QSAR predictions for many years and therefore the prediction of logP has been examined countless times. One of the best performing models is to predict the logP using multiple methods and average the result. We have used those averaged predictions to develop a training-set which was able to distil the information present across the disparate logP methods into one single model. Our model was built using extendable atom-types, where each atom is distilled down into a 6 digit number, and each individual atom is assumed to have a small additive effect on the overall logP of the molecule. Beyond the simple coefficient model a consensus model is evaluated, which uses known compounds as a starting point in the calculation and modifies the experimental logP using the same coefficients as in the first model. We then test the performance of our models against two different datasets, one where many different models routinely perform well against, and another designed to more represent pharmaceutical space. The true strength of the model is represented in the pharmaceutical benchmark set, where both models perform better than any previously developed models.

**Electronic supplementary material:**

The online version of this article (10.1186/s13321-018-0316-5) contains supplementary material, which is available to authorized users.

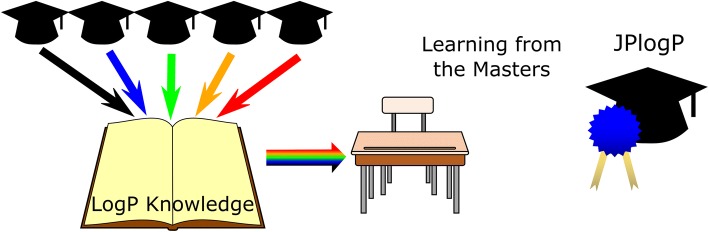

## Introduction

The log of the partition coefficient between octanol and water (logP) is a widely used descriptor in a multitude of QSAR problems. A compound’s logP is used in numerous different QSARs and QSPRs for the prediction of many different activities and properties. It has been used for skin penetration, as exemplified in the Potts and Guy equation [1], as well as Lipinski’s rule of 5 [2] for determining drug likeness. It has also been used to estimate solubility [3] and has been deemed important for the prediction of general toxicity towards aquatic animals [4]. These wide-ranging uses show that it is an important property, applicable to a number of different biological outcomes related to permeability and accumulation, and that accurate predictions are a necessity. As such, there is a vast quantity of prior research towards providing reliable and accurate predictions.

There are numerous methods for the prediction of logP, but they essentially fall into two major classes. They either use a contribution method, where atoms or fragments contribute a portion of the whole molecules logP, or a property-based QSAR for predicting a value. A recent review on the subject has highlighted the difficulty in comparing the performance of different methods, because the methods are nearly always trained on one dataset, the PhysProp database [5], which is the best public source of experimental logP data. This means that the performance of each model was optimised for the chemical space of the PhysProp dataset, which isn’t necessarily the chemical space of interest for every application. Mannhold compared the performance of many different logP calculators to an in-house Pfizer dataset [6] and found that a very simple equation was able to predict a compound’s logP with an accuracy comparable to that of many of the better prediction methods. It performed especially well considering it had only two parameters; the number of carbons and the number of heteroatoms. As Pfizer is unlikely to release its database of 96,000 chemicals along with their logP values, another benchmarking dataset has kindly been gathered by Martel [7]. This study experimentally measured the logP of 707 compounds, using an HPLC method, and released it as a benchmarking dataset for use within the community. This dataset consists of compounds that are more consistent with a typical pharmaceutical company’s chemical space compared to the PhysProp dataset, and as such is a more appropriate test set to use as a benchmark.

When investigating the contribution methods one notices that they are further subdivided into larger fragment methods and atom-typer methods. In each case the structure is split into pieces and each piece has a fractional contribution to the overall logP of the entire molecule. The major difference between these two types is solely the size of the fragment. The actual fragment methods use fragments that are larger than a single atom, while the atom-typer methods are when each fragment consists of a single atom. Typically, the atom-typer methods also incorporate further structural information such as the hybridisation and the atoms that are bound to the atom thereby keeping some of the additional information that is found in the fragment methods. This has been a very fruitful area of interest over the years and has generated many of the popular logP methods such as XlogP2 [8], XlogP3 [9], AlogP [10], SlogP [11], AlogPs [12], ClogP [13, 14], and ACDlogP [15].

The other major class of logP prediction relies on the use of standard QSAR by calculating descriptors for the molecule and then using those descriptors to predict the logP. There are varying levels of complexity for the descriptors, running from simple counts of carbon and heteroatoms [6], to attempts to correlate the solvation energies of molecules with complicated calculations to predict their logP value [16]. A commonly used method of this type is MlogP [17] from Moriguchi, as it is built on well-described descriptors that are also easy to calculate. For further information on the comparison and benefits of the various methods, see the extensive paper by Mannhold [6].

As each different prediction method will predict slightly different logP values for any given compound, it was thought that a potential way to develop a new training set would be to use multiple different logP prediction methods on a large diverse dataset leveraging the knowledge present in each individual predictor. Tetko et al. have successfully built models trained on predicted data to allow transfer of knowledge from corporations without revealing the secure data underlying the knowledge [18]. Mannhold et al. built a consensus logP model, which was the simple average of all of the best performing models, and it was the best predictor across all of the private data. It logically extends that building a training set on averaged predicted values could also capture that performance. It is hoped that the resultant model will provide a better prediction, akin to the improvement that is possible when combining multiple models into an ensemble model. In essence we hoped to capture the knowledge available in many different models and distil it into one simple model by training using the predicted data [19].

## Methods

**Fig. 1:**
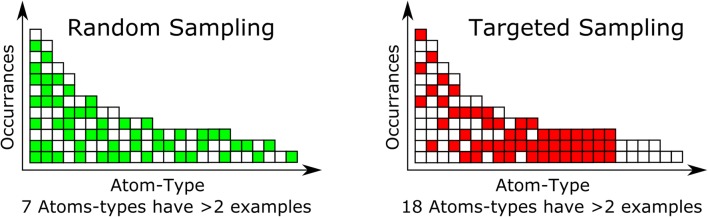
Random sampling versus targeted sampling. If there is a minimum learning requirement of 3 examples the random only is able to use 7 columns, while the targeted is able to learn on 18 columns

We used the averaging leveraged in the consensus model to generate the training set. We had selected the NCI-DB [20] as having a good coverage of the important chemical space for many different industries. This was still too large a dataset to use for training (~ 260 k compounds) so a targeted sampling method was used to produce a dataset with maximal diversity of different atom-types while still using a moderate number of compounds. This was accomplished in KNIME [21] (along with some internal java code) by first generating a hologram for each compound, consisting of the count of each atom-type, and then by identifying the total number of occurrences of each atom-type in the dataset. This occurrence list was sorted in increasing quantity and then atom-types with less than three occurrences were removed, due to insufficient information for training potentially resulting in spurious coefficients resulting from overfitting rare atom-types. Then, in an iterative process, the dataset was searched for the atom-type in question and the compounds that had that atom-type present were selected for the training set. The process is described in Fig. 1 with a sampling level of 3. To generate our training set we used a sampling level of 1000, meaning that, once all the rarer atom-types were collected, if there were more than 1000 compounds with a given atom-type then the compounds were sampled randomly to select a maximum of 1000 compounds per atom-type. We then went on to the next fewest occurrence atom-type until we reach the most occupied atom-type. This allowed for the selection of all of the compounds with the rarer atom-types, while still allowing for the adequate sampling of the common atom-types without the risk of not randomly selecting a rare atom-type and thereby having a prediction that would be classified as out of domain.

With this diverse compound set, containing 89,517 compounds (see Additional file 1), in hand we obtained logP values from four different calculated logP methodologies: AlogP [10] (from Vega), XlogP2 [8], SlogP [11], and XlogP3 [9]. These methods were chosen as they were either open source and therefore freely available, or used after having received the authors permission. They are also in the top half of the performance metrics against the Martel dataset. Previously the use of calculated data has been used to obfuscate the training compounds enabling secure data sharing [21], but we are attempting to consolidate the knowledge across multiple models into one. The accepted value for each compound was calculated as the arithmetic mean of the values from the four methods. This overall consensus logP is an attempt to mimic the consensus values that performed quite well in Mannhold’s datasets. By using this calculated dataset, we hoped to be able to distil the knowledge present across multiple models down into one new model, thereby leveraging the information from each model into a single prediction [19]. Each individual model would act as a teacher to our model. As a result we hoped to improve the accuracy in the prediction by using all of the knowledge available in each method, and in essence generate an average prediction without the necessity of running multiple predictors.

**Fig. 2:**
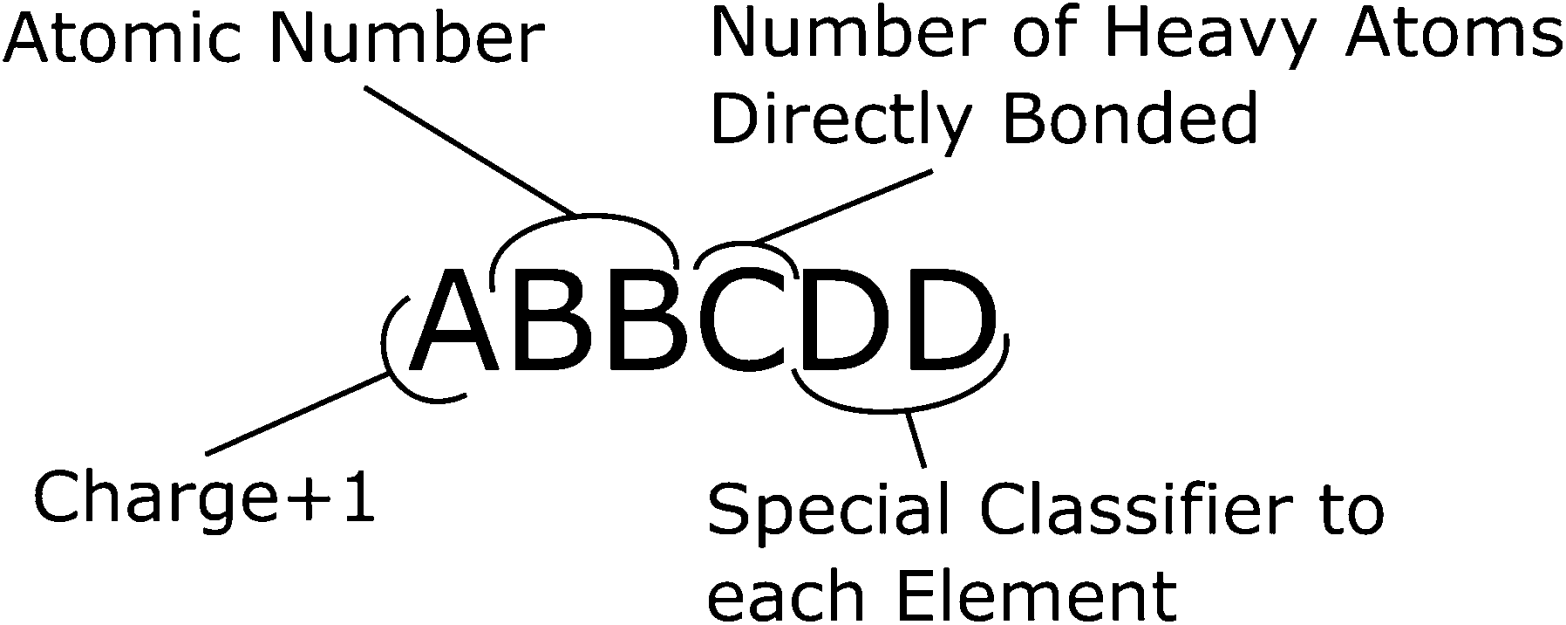
Number format of the identifier for each atom in the atom-typer

**Fig. 3:**
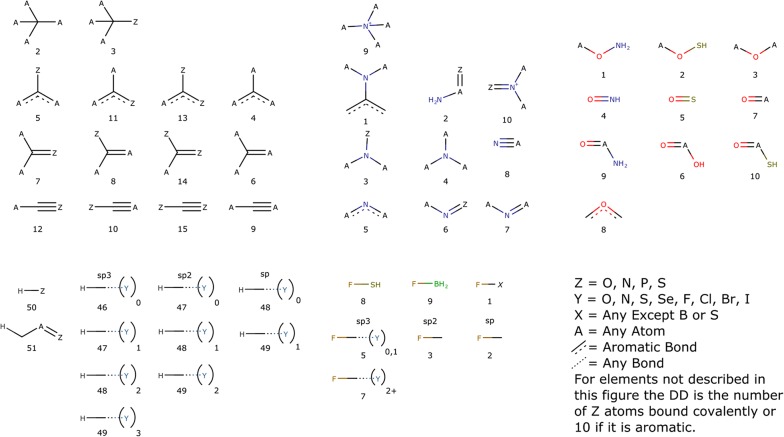
Special codes for the local environment around the atom being typed

The atom-typer developed for this paper is organised so that each atom can be represented by a six digit number as defined in Fig. 2. The first digit, A, is the charge plus one, to keep the numbers positive, while still allowing for atoms with a formal negative charge. In organic chemistry space it is very unlikely that there would be an atom with a charge of − 2. The next two digits, BB, are the atomic number, thereby enabling the separation of each atom-type by element. As this is fixed at two digits, atoms with an atomic number greater than 99 are incompatible with this atom-typer. Since we are concerned with organic compounds and not of organometallic complexes, this isn’t really a hardship. The properties of organometallic compounds are governed significantly by their counter-ions and are outside the scope of this method. The fourth digit, C, is simply the number of non-hydrogen atoms bound to the atom being typed, in an attempt to capture steric information. The last two digits, DD, are specific to each element and are detailed in Fig. 3. They relate to the hybridisation of each atom as well as containing information about the neighbours near the atom being typed.

The special classifiers, DD from Fig. 2, relate to the local environment surrounding the atom being typed. These are broken down by element, with specific cases for carbon, nitrogen, oxygen, hydrogen and fluorine, but with a default case for all other elements. In the case of carbon, the special classifier is divided into four groups based on the maximum bond order, and those groups are further divided based on the number and location of polar atoms bound. For the purposes of this atom-typer, a polar atom is either a sulphur, oxygen, nitrogen or phosphorus atom. Carbons hybridised as *sp*3 were only divided into two groups depending on whether a polar atom is bound or not. However with the higher bond order cases (aromatic, double or triple) then the location of the polar atoms is important as a multiply-bound polar atom will change the properties of a carbon atom in a different manner than a singly-bound polar atom, for example the carbon alpha to a pyridine experiences a different pull compared to the carbon of an aniline, although in each case the carbon is bound to only one polar atom. In the case of nitrogen the effect of substituents on the lone pair is considered to be more significant than the effect on the absolute hybridisation, by including a special case to account for aniline- and amide-like compounds as the lone pair is less available for interaction with water in those types of molecules. As with carbon, there are also special classes for nitrogen directly bound to a polar atom. For oxygen, better results were obtained by being more specific than a simple polar atom bound motif. Instead we have specified the special cases to nitrogen and sulphur directly, rather than combining them into a monolithic entity, as done for nitrogen and carbon. Also separated out are the oxygen atoms in amides, acids, esters and their thio equivalents as they all have different reactivity with regards to the *sp*2 oxygen, so it made sense to keep those atom-types separate. For fluorine, again the classes mostly cover the atoms it is bound to, but an important consideration is the hybridisation of a carbon it is bound to, as well as any additional electron withdrawing substituents on that carbon. As fluorine is very electron withdrawing, it can have an inhibitory effect on other electron withdrawing substituents, and accounting for this increased the performance of the model. The other halogens were treated in the default manner, as their withdrawing strength is more on par with Nitrogen as measured by their electronegativity. Hydrogens are treated very similarly to the method that Ghose and Crippen developed [10], with the exception of removal of the special cases for polar atoms on carbons bonded to the carbon that carries the hydrogen. As a default any atom not fitting into the previously described categories is differentiated by if it is an aromatic atom, and otherwise differentiated by the total number of polar atoms bound. Overall, this method is able to identify and isolate atoms with differing chemistry and in differing environments and has been tuned to account for differences in electronic structure to capture the interactions between the compound and solvent.

The model was trained by generating an atom-type hologram of each compound in the training set. A hologram is similar to a fingerprint, but contains the count of the occurrences of each atom-type, not merely their presence or absence. The holograms are then subjected to a multiple linear regression to find the coefficients necessary to solve the amount that each atom-type will impact the logP of each molecule. This was accomplished by QR-Decomposition using the JAMA matrix algebra package in Java [22]. The matrix solved with a R^2^ value of 0.975, showing that the coefficients are capturing nearly all of the required variance present in the dataset. If the model is used to predict the training set the RMSE is 0.362. Once the coefficients have been determined, then a prediction is as simple as generating the atom-type hologram and multiplying through the hologram with the coefficient vector to generate a coefficient-only, calculated logP, called herein: JPlogP-coeff. These coefficients are provided in Additional file 2. This method uses a standard assumption that the logP of a molecule can be determined as the sum of the logP contributions of all of the atoms present in the molecule. This assumption underpins every atomic and fragment contribution method.

1 $$^{''} JP\log P - coeff^{''} = \sum\limits_{atom - type}^{n} {\alpha_{n} *Count_{n} }$$

Equation 1: Summation showing how the prediction is generated. The count of typed atoms is multiplied by the coefficient calculated from the least squares fitting. This is summed over all of the atom-types present in the molecule to generate the predicted logP value.

To attempt to improve the performance an additional prediction method was investigated. This involved using an ensemble prediction using available knowledge in existing experimental logP values. This allows us to use a known value as a starting point, if the compound to predict is similar to the known compound. The similarity measure used was a Tanimoto-like measure, but as the molecules are represented by counts, instead of bits, the value of each position was set to the minimum occupancy divided by the maximum occupancy. If a molecule with a known experimental logP passes the similarity threshold, then it can be used as a known starting compound in the calculation of logP for the unknown compound. For a graphical representation of this process see Table 1. As the model is only dependent on the count of all of the atom-types in the molecule, without any connectivity information, then this is simply achieved by using a difference hologram. We initially take the atom-type hologram of both the unknown and the known compounds. We then subtract the known from the unknown generating a difference hologram that contains the difference in atoms-types between the two molecules. This difference hologram is also multiplied by the coefficients of each atom-type and the result is added to the logP of the known compound, resulting in the predicted logP of the unknown compound. Starting from a similar compound with an experimental logP value has the effect of removing the imprecision inherent in the least squares solution by minimising the number of coefficients required. For example, if you wanted to know the logP of bromophenol and knew the logP for chlorophenol, the overall change is subtraction of the portion that represents a chlorine and addition of the portion that accounts for the bromine (Table 1 and vide infra). We used the PhysProp database of logP values as our library of compounds to search, but if desired it is possible to add compounds to the library to improve predictions. This allows for a user to improve the performance of the model by tuning it to their section of chemical space.

**Table 1:**
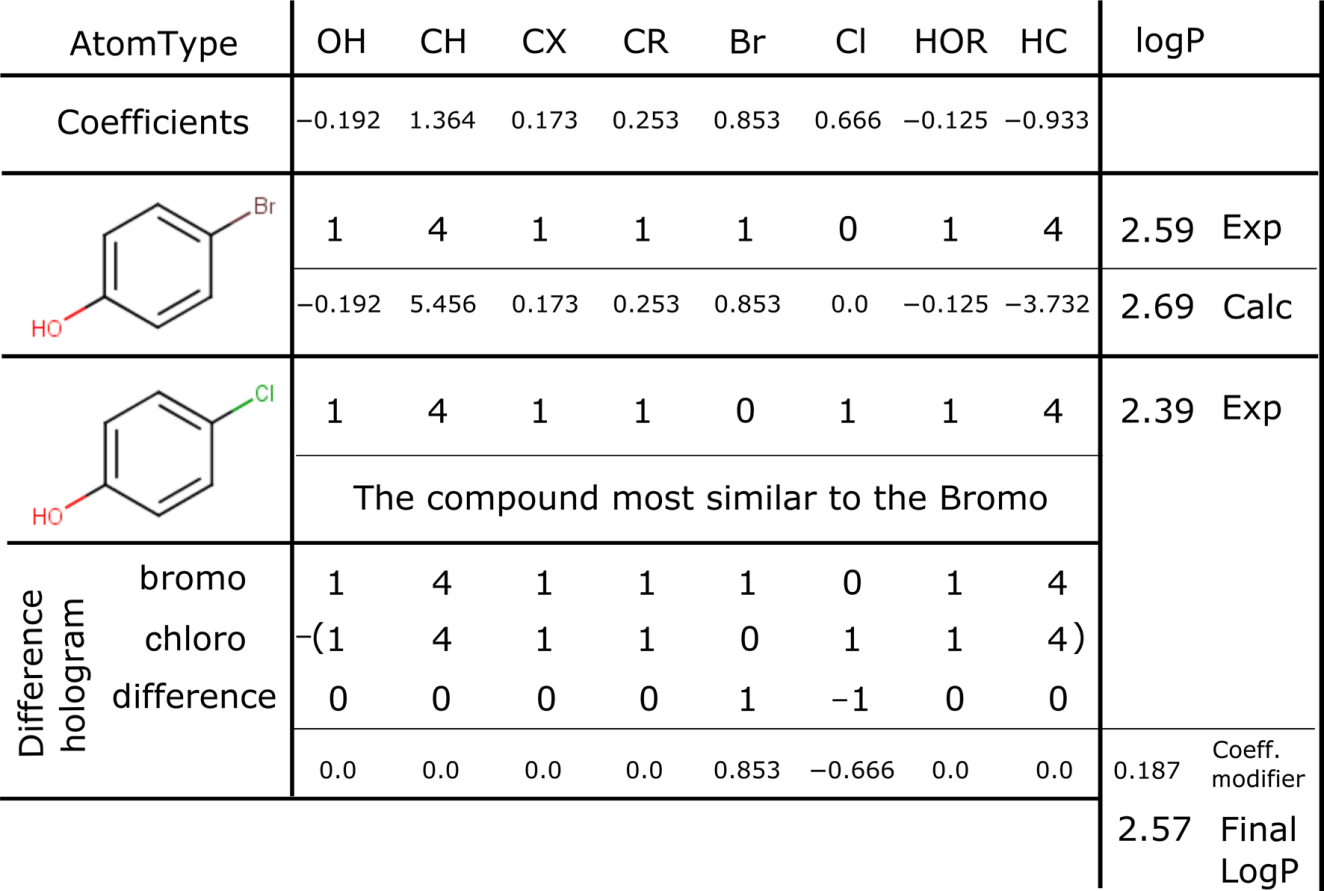
An example calculation showing both JPlogP-coeff and JPlogP-library

## Results and discussion

After the atom-typer and the training set were finalised and all the code was implemented in our in-house cheminformatics engine we examined the performance of our predictor against the performance of different publically available logP prediction methods. We used two benchmark test sets. The first was provided by Avdeef [23] and consists of a large number of compounds present in the PhysProp database [5]. The second, which was provided by Martel [7], was chosen to represent the more difficult pharmacologically relevant chemical space. The use of that test set also avoids problems associated with compounds that are in the test set also being present in the training set. Our results are split between our two different prediction methodologies: JPlogP-coeff and JPlogP-library. The first relies solely on the coefficients trained from the model, while the latter starts from similar compounds to the compound being predicted and instead only uses the coefficients to modify the known logP to represent the difference between the molecules.

As an example, a sample calculation is shown in Table 1. This shows the atom-type holograms for two molecules, 4-chlorophenol and 4-bromophenol. Also given are the coefficients for the different atom-types. It can be seen that using solely the coefficients, the prediction for bromophenol is 2.69, which is within acceptable limits from the experimental logP value of 2.59. Instead, if one starts with the experimental logP of chlorophenol of 2.39 and determines the difference hologram between the known chlorophenol and the theoretically unknown bromophenol, you can then use the remaining coefficients along with the initial logP from chlorophenol to arrive at a library prediction of 2.57. That result is significantly closer to the experimental value of 2.59 than compared to using only the coefficients to predict the logP. This method is similar to the way XlogP3 incorporated additional knowledge via a library of known compounds into their predictions, except we are able to start from more than one similar compound, but as the compounds are compared by hologram only there is the possibility of more major structural differences.

A further optimisation was required to determine what the minimum level of similarity should be and also the number of similar compounds that can be used for a prediction. The Avdeef [23] dataset was used to optimise these parameters, as that was closer in chemical space to the PhysProp database. The similarity measure used was similar to the Tanimoto measure, but allowed for partial overlap by using the minimum value divided by the maximum value for each bit instead of a simple one or zero. The difference hologram method works best when starting from similar molecules, so no similarities below 0.5 were tried. The averaging of many different calculations was considered unnecessary, so using 7 different best matches was deemed to be the maximum. Shown in Table 2 are the results of the different combinations tried. The minimum similarity for a match to be possible was varied between 0.5 and 0.8, along with the maximum number of potential matches varying from 1 to 7. The optimised parameters resulting in the minimum root mean squared error (RMSE) for the dataset were found to be a minimum similarity of 0.75 along with a maximum number of 5. If the coefficient method is used, without using the extra information in the library, the RMSE is 0.808. Given that the minimum similarity is set so high, it is a rare event that there are the full five initial matches used for any given prediction, but with a more highly tuned personal library the possibility exists.

**Table 2:** RMSE values for the grid-search comparing the maximum number of matches to use along with the minimum similarity threshold

Maximum examples	Minimum similarity threshold				
	0.8	0.75	0.7	0.6	0.5
1	0.65	0.647	0.659	0.704	0.731
2	0.644	0.639	0.651	0.663	0.687
3	0.642	0.636	0.645	0.656	0.678
4	0.64	0.634	0.644	0.654	0.677
5	0.639	0.633	0.643	0.651	0.672
6	0.639	0.633	0.642	0.649	0.67

We also compared the ability of two alternative atom-typer methods to capture the knowledge in the calculated logP dataset. We compared both Sybyl and the original Ghose and Crippen atom-types. The sybyl atom-type only contains 44 different possibilities, which was extended to include all of the possible metals as their own individual types. Using the calculated training set only 28 different atom-types were found, but the resultant model still predicted the Martel benchmark set with a respectable RMSE of 1.15. The Ghose and Crippen atom-types are an expanded set and using our calculated training set 108 different atom-types are found and the model built using these, was able to improve on the sybyl result yielding an RMSE of 1.12. We also investigated the potential of using XlogP2 atom-types, which resulted in a model with 88 types and a RMSE of 1.17. With the atom-typer defined herein, 188 different atom-types are found and the result improves to an RMSE of 1.04, using only the coefficients to produce a prediction. The added complexity is better able to account for minor variations and with the large calculated datasets and are better able to predict across a wide ranging chemical space. When examining the impact of each additional prediction method being averaged into the calculated value on the performance against the Martel dataset a clear trend is seen for each additional methodology. Starting from the best predictors when XlogP3-AA and SlogP are used as the sole contributors to the calculated dataset the performance has a RMSE of 1.08. With the addition of AlogP (Vega) it is reduced to 1.073. Finally the addition of XlogP2 yields the performance of 1.04 RMSE. It is likely that the additional incorporation of Molinspirations logP and Biobytes logP could result in an improvement, but that would require licensing the models and is outside the scope of this project. The addition of the next best performing model, AlogPs, resulted in a minor decrease in performance to 1.088 so the addition of further models ceased with the averaging of four. This trend is also seen when simply averaging the results from each individual model. When XlogP3-AA and SlogP are averaged together the RMSE against the Martel test set is 1.159. With the addition of AlogP it falls to 1.149 and the addition of XlogP2 drops it further to 1.123. This result is detailed in Table 4 as LogP4Average. Again when AlogPs is added to the average the resulting combined model performance decreases slightly to a RMSE of 1.136. It appears that by averaging the results to generate the training set and then learning with a more complicated model results in better performance.

The strength of using the calculated data as opposed to experimental data is apparent when attempting to train JPlogP using the PhysProp database. The JPlogP atom-typer is exceptionally data hungry though and having such a large number of parameters means it requires a large volume of data to avoid overfitting. This works well in conjunction with the use of calculated data as there is no limit, other than the available memory in the computer, to the amount of data to feed into the method. Conversely when using experimental data when trained using the PhysProp database alone the R2 of the solution dropped to 0.884 on only 92 indices with a RMSE of 1.17 against the Avdeef dataset and 1.35 against the Martel dataset.

To evaluate the performance of our model we initially tried to locate every logP methodology tried by Mannhold, but a number of them required a license or were currently unavailable. Instead we settled on the reduced set detailed below where they are freely available within KNIME, can be simply accessed via a web interface, or can be run one compound at a time for free. As there are only 707 compounds in the Martel dataset and 267 compouonds in the Avdeef dataset it was possible to run each compound manually. In the end we selected the following list: ACD logP [15], AlogP (Vega) [24], AlogP (CDK) [25], AlogPS [12], Biobyte ClogP [13], KowWIN (EPISuite) [26], Mannhold logP (CDK) [25], Meylan (Vega) [24], MlogP (Vega) [24], MolInspiration logP [27], SlogP (RDKit) [28], XlogP2 (CDK) [25] and XlogP3 [9].

Initially, we investigated the performance of our method and a subset of other methods (Table 3) against the public dataset gathered by Avdeef [23]. This is quite similar to the public dataset used by Mannhold. Here, the advantage that is given to the predictions by using the library lookup of known compounds is immediately apparent. As a large number of compounds in the dataset are exact matches, the RMSE is quite low at 0.63. When using JPlogP-coeff the error rises to 0.81, still performing broadly similar to all of the easily available logP predictors. The performance of LogP4Average is better than the four models that were averaged together, as also occurred in Mannholds analysis of this dataset. The second best is AlogP from Vega, followed by Biobyte’s ClogP then XlogP3-AA. Mannholds method comes in at 1.43 RMSE, which is respectable performance for a very simple model with just two parameters. The bulk of the methods are broadly similar around the 0.8 RMSE range. All of the models perform better than the average of the values in the dataset, herein designated the Arithmetic Average Mean (AAM), with the sole exception of AlogP from CDK, as implemented in KNIME. As this is so different from the AlogP implementation in VEGA there are suspicions that the poor results are due to either an error in the implementation or a user error. Trying varying methods of standardisation, i.e. all combinations of aromatisation/dearomitisation and with explicit/implicit hydrogen treatment did not improve the results so they are left more as a curiosity than to be considered a proper result. Our method reaches an average error of approximately 0.6 log units, which is only double the experimental error discovered in a study of repeated measurements of the same compound [16].

**Table 3:** Performance of different logP methods against the Avdeef dataset

Predictor	Performance	%Binned absolute errors				
	RMSE	< 0.5	0.5–1	1–1.5	1.5–2	≥ 2
JPlogP-library	0.63	68.91	20.6	7.12	2.25	1.12
LogP4Average	0.65	71.91	16.11	7.49	3.37	1.12
AlogP (Vega)	0.65	70.04	18.35	6.74	3	1.87
Biobyte CLogP	0.76	70.41	17.23	4.12	3.75	4.49
XlogP3—AA	0.77	69.29	15.36	8.24	3.75	3.37
SlogP	0.79	49.06	34.46	10.49	4.12	1.87
Molinspiration	0.80	63.30	20.23	10.49	3.37	2.62
JPlogP-coeff	0.81	47.94	32.58	13.48	4.49	1.5
ACD	0.83	68.17	19.10	8.24	1.87	2.62
KowWIN	0.84	73.78	14.97	5.99	2.25	3.00
MlogP (Vega)	0.85	67.04	16.85	6.74	5.24	4.12
AlogPS logP	0.86	66.29	23.60	7.12	2.25	0.75
Myelan (Vega)	0.89	65.54	15.73	9.74	4.49	4.49
XLogP2	1.05	56.93	20.22	8.99	7.12	6.74
Mannhold LogP	1.43	26.22	24.72	20.97	13.86	14.23
AAM	1.62	21.35	23.97	18.73	12.73	23.22
AlogP (CDK)	2.57	7.87	10.49	19.1	14.61	47.94

However, a major problem with using this dataset as a test set is that most of these methods used a large portion of the test set as their training set. In fact, the major increase in performance in our model is simply down to the fact that the library of reference compounds contains exact matches and therefore isn’t predicting the logP value as much as it is just remembering what the logP value is from a table.

A much more interesting challenge, therefore, is the prediction of the Benchmark dataset devised by Martel; the performance of a number of logP predictors against the Martel test set [7] is shown in Table 4. Detailed results are available in Additional file 3. Every predictor selected for this study was able to generate a prediction for every compound. This shows the effort and quality of the benchmark dataset provided by Martel. Every structure is curated to a high standard and is able to be understood by each program in turn. The dataset is also quite a difficult one to predict, as the fourth best predictor is the simple Arithmetic Average Mean (AAM) result which is simply the average of every known logP value in the test set (4.189). The performance of most of the predictors is broadly similar with an RMSE between the prediction and the experimental value of approximately 1.3 log units. JPlogP has the best performance of all of the different methods used. The experimental values range from 0.3 to 6.96 so they are over a rather narrow range and, furthermore, are more or less a normal distribution. If the range were larger, or the distribution broader, then the performance of the AAM would decrease and would show more of the predictors in a positive light. A good enhancement to this benchmark set would be some additional compounds at the more extreme ends of the dataset, which would make it more difficult for the AAM to predict with good results as over half the dataset is within one log unit of 4.189. By broadening and extending the distribution, the relative performance of the AAM would decrease allowing for the strength of the various predictors to be more apparent.

**Table 4:** Performance of different logP methods against the Martel dataset

Predictor	Performance	% Binned absolute errors				
	RMSE	0–0.5	0.5–1	1–1.5	1.5–2	≥ 2
JPlogP-Coeff	1.04	39.7	31.5	14.7	7.3	6.6
JPlogP—library	1.05	38.6	32.4	14.9	7.4	6.7
LogP4Average	1.12	33.9	29.4	19.2	10.8	6.6
XlogP3-AA	1.16	33.0	27.9	20.7	10.5	8.1
AAM	1.18	34.2	27.6	17.5	11.7	8.9
Molinspiration	1.21	32.7	26.3	20.1	11.7	9.2
SlogP	1.24	32.4	27.6	19.8	10.7	9.5
ALogP (Vega)	1.24	34.4	27.4	17.5	9.8	10.9
Biobyte CLogP	1.24	32.0	31.3	16.8	9.1	10.9
XLogP2	1.28	31.8	24.5	19.9	11.6	12.2
AlogPS logP	1.29	25.3	28.3	20.1	16.3	10.0
ACD	1.39	33.1	25.2	17.5	10.6	13.6
KowWIN	1.42	32.0	23.2	18.2	11.9	14.7
Meylan (Vega)	1.66	25.7	23.2	17.1	14.3	19.7
Mannhold LogP	1.71	14.1	16.3	23.2	20.9	25.5
MLogP (Vega)	1.95	12.3	14.7	17.7	19.5	35.8
AlogP (CDK)	3.72	1.6	2.5	5.0	9.1	81.9

When looking at the absolute errors in the predictions a general trend can be seen, where the predictors with the lowest errors have the predictions that have the lowest absolute error, with some exceptions. Both ACD and KowWIN have a RMSE higher than AlogPS, but oddly both of those have more compounds with less than 0.5 log unit error than the AlogPS model. JPlogP is able to give a prediction to within one log unit approximately 70% of the time, which again leads the pack. The worst performing, in terms of error and RMSE was again the AlogP from CDK (vide supra). Overall most of the different logP methodologies perform worse on the more difficult Martel dataset than they did on the Avdeef dataset, mostly because the Avdeef dataset is significantly closer in chemical space to the training set used by the different methods.

## Conclusions

We have developed a new atom-typer and applied it towards the prediction of logP using an atomic contribution method. This was trained on a dataset of multiple predicted values averaged together in an attempt to concentrate the knowledge present across multiple logP predictors. The model was applied to both a commonly used and a completely external test set. The performance of JPlogP was comparable to the other models examined with the commonly used test set, but was an improvement over the others against the external benchmarking set. Importantly, there were no compounds considered out of domain, showing that a sufficiently large training set had been used. The atom-typer methodology developed should be applicable to other cheminformatics problems where there is both a sufficiently large training set and the solution of that problem relies on contributions of different atoms to the whole property. The performance improvement is incremental, as the prediction of logP is a well-established field, but nonetheless these results should hopefully provide improvement to the use of calculated logP parameters in the future. JPlogP will be available as a KNIME node from Lhasa Limited in the trusted community contributions in due course, as well as a descriptor within CDK (See Additional file 4). The version released will be based on CDK instead of our in-house platform, which has slightly improved performance (1.04 in house RMSE vs 1.01 CDK RMSE) against the Martel dataset but the platform is open source and as such is available for release without any licensing agreements. There are also fewer atom-types in the CDK model (188 vs 175, see Additional file 5) and the coefficients are different, but the difference in prediction between the two models is minimal with over 85% of the difference between the predictions being less than 0.3 of a logP unit. The different versions just found different least squares solutions, but the predictive power between them is essentially the same.

## Additional files

**Additional file 1**. The calculated training set used in model generation.

**Additional file 2**. Trained coefficients for the JPlogP model.

**Additional file 3**. Results of the tested logP methods against the Martel dataset.

**Additional file 4**. Source code for the CDK implementation of JPlogP.

**Additional file 5**. Occurrances of each atom-type in the training dataset.

## Abbreviations

AAM: arithmetic average mean
CDK: chemistry development kit
RMSE: root mean squared error
QSAR: quantitative structure activity relationship
QSPR: quantitative structure property relationship
